# Book Review

**Published:** 1934

**Authors:** 


### Reviews of Books

**The Clinical Examination of the Nervous System.** Sixth Edition. By G. H. Monrad-Krohn, M.D. Pp. xix., 234. Illustrated. London: H. K. Lewis & Co. Ltd. 1933. Price 7s. 6d.—We have reviewed this book on several occasions previously in these columns. It has now, however, reached its sixth edition, which proves that it is a work which has been much appreciated and one which is most useful to students, house physicians and general practitioners. Numerous minor additions and alterations have been made in this edition, more particularly in various methods which are of clinical importance, including Laruelle's method of encephalography.

**The Adrenal Cortex.** By L. R. Broster, O.B.E., D.M., M.Ch., and H. W. C. Vines, M.D. Pp. 94. Illustrated. London: H. K. Lewis & Co. Ltd. 1933. Price 6s.—This book describes in detail a type of sexual disturbance which has now been crystallized into a well-established clinical syndrome, the so-called "Adreno-Genital Syndrome," in which certain lesions of the adrenal cortex have been associated with hirsutism and virilism in women. Why apparently similar pathological lesions should be associated clinically sometimes with this syndrome and sometimes without it has not been adequately explained. The authors, however, claim to have found a differential stain (Ponceau Fuchsin) which has given positive results in all their cases subjected to unilateral adrenalectomy, and which enables them to separate the two types, such as simple hyperplasia, cortical adrenoma, cortical carcinoma or hypernephroma. The authors consider that there must be some special peculiarity of the cortical cells in virilism, involving the production or over production of a specific secretion. The fuchsinophil material which they describe appears to be related to the masculinizing function of the cortex, a function which is probably physiologically normal, but which in "virilism" is exercised to excess. The clinical condition of eighteen cases is described in detail. The book also contains many interesting observations, and coloured plates showing the condition when the reaction is present and also when it is absent.

**Dermatology and Urology.** By FRED WISE, M.D., and MARION B. SULZBERGER, M.D. The Practical Medicine Series. 1931. Pp. 458. Chicago: The Year Book Publishers. This handy little book contains abstracts from the dermatological and urological literature of the world during 1931. It is a publication more suitable for the specialist than the general practitioner, though the authors state they desire to help the latter. The first half of the book is devoted to abstracts from an immense field of recent articles on dermatology, whilst the second half is given up to a very comprehensive survey of current literature in the domain of urology. The majority of the abstracts are enriched by a little editorial comment and criticism. Those engaged in these branches of special medicine will find a most valuable addition to their knowledge in this splendidly brief and concise book.

**Massage and Remedial Exercises.** Second Edition. By NOËL M. TIDY. Pp. xii., 430. Illustrated. Bristol: John Wright & Sons Ltd. 1934. Price 15s.—The fact that a second edition of Miss Tidy's text-book has been found necessary within eighteen months of the first is a tribute to its having filled the void for which it was intended; this, the author explains, is to provide for senior students of massage and those recently qualified, for whom other books on massage offer too few details; while the advanced works, like Dr. Mennell's, could not be appreciated without "a far more extensive background of knowledge and experience than would be possessed by any medical gymnast at the beginning of her career." The wealth of detail provided by Miss Tidy certainly offers the student an opportunity of acquiring such a background, if she is not stunned by its complexity; for one cannot help feeling that the average intellect entering such an area is apt to lose sight of the wood for the trees. Text-books such as these are inevitably designed to meet the demands of qualifying examinations, and certainly the one under review is a model of accuracy and up-to-date information, representing the digestion of an enormous amount of information and the teaching of many schools. Nevertheless, one feels certain that the practitioner of massage, like that of medicine, would be safer if thoroughly grounded at the outset of her career in certain fundamental principles, which are applicable to all joints and all diseases, and left to discover their modifications and the subtleties of practice by the actual handling of cases. If, on the other hand, the training schools demand the minutiæ contained in this text-book, we would strongly recommend the publishers to re-issue it in a set of handy pocket volumes with a print that would not strain the eyes of those whose bookwork is done at the end of a day of strenuous exercise, which has already lowered their muscle tone.

**Aids to Operative Surgery.** Second Edition. By CECIL P. G. WAKELEY, F.R.C.S., D.Sc. Pp. viii., 225. London: Baillière, Tindall & Cox. 1934. Price 3s. 6d.—There is a marvellous amount of information packed into this little book, and it is very cheap. Gynæcological, ear, nose and throat operations are included. For students taking a class in operative surgery it is excellent, they need nothing better. It contains all they are likely to be asked in a surgical examination for a pass degree; indeed, no examiner has any business to expect a tithe of what is here contained of operative detail. The author's technique is not always that most favoured to-day; we think Coffey's method of transplanting the ureters for ectopia vesicæ might well be substituted for the three procedures mentioned; it is customary to stretch or divide the sphincter for anal fissure; an orchidopexy is more likely to be successful if some method of holding the testis down is used. The treatment described for exophthalmic goitre is misleading. As the author devotes a paragraph to discuss means of checking hæmorrhage from the superior longitudinal sinus, it might well be mentioned that a piece of the temporal muscle stitched over the tear is simple and effectual. The book is well turned out.

**Mayou's Diseases of the Eye.** Fourth Edition. Edited by F. RIDLEY, M.B., and A. SORSBY, M.D. Pp. xvi., 249. Illustrated. London: Oxford University Press. 1933. Price 6s. **Student's Guide to Fundus Appearances.** By F. RIDLEY, M.B., and A. SORSBY, M.D. Pp. 16. Illustrated. London: Oxford University Press. 1933. Price 2s. 6d.

**Mayou's Diseases of the Eye.**—This book must surely be so well established in the affections of students as to need no introduction. Since the appearance of the last edition, however, a great deal of new work has been published. This the authors have carefully sifted, and much that seems likely to be of permanent value has been incorporated in the present edition. This has meant the re-writing of much of the book, but its essential characteristics remain the same, and, within a small compass, it succeeds in covering adequately the whole field of ophthalmology. That this is accomplished without the sacrifice of clarity of style and without obvious condensation of subject-matter is a tribute to the authors. Some of the views expressed—*e.g.* on glaucoma—are a little in advance of those commonly taught, but this is probably good for both students and teachers. Altogether, this is a book which should be of great value to students and general practitioners. **Guide to Fundus Appearances.**—In order to reduce the cost of the book the usual coloured plates, illustrative of fundus appearances, are published separately in a small volume, in which are a dozen plates, some of them of composite character. With its descriptive letterpress it furnishes a fairly complete guide to such fundus conditions as the student may be expected to recognize.

**Miners' Nystagmus.** By F. O'SULLIVAN, M.B. Pp. 100. Illustrated. Bristol: John Wright and Sons, Ltd. 1933. Price 5s.—It is probably not generally realized, outside the large mining areas, how much incapacity results from the somewhat ill-defined condition known as miners' nystagmus, nor that, as yet, very little advance in its prevention has been made. It is in order to stimulate others to carry out research on the subject that the author has written his monograph. After a brief survey of nystagmus in general, he passes to a consideration of the symptoms, physical signs, etiology and treatment of miners' nystagmus, and exemplifies his views in a series of fifty case reports. This is a book which should interest those medical men whose work brings them in contact with the disease.

**Modern Advances in Diseases of the Throat.** By A. MILLER, F.R.C.S. Pp. xii., 120. Illustrated. London: H. K. Lewis & Co. Ltd. 1934. Price 10s. 6d.—The title of this book is misleading. In the first place its scope is limited to disease of the tonsils alone; in the second it contains very little that can claim to be "modern advances." Indeed, if we mention Brown Kelly's researches on tortuosity of the carotid, a short account of agranulocytosis and diathermic treatment of the tonsils, we have practically exhausted the list. After a full description of the accepted anatomy of the tonsil the bulk of the book is devoted to a tedious *réchauffée* of indications for and technique of tonsillectomy. Complications are discussed at length, though not their treatment, except that of hæmorrhage, and even here the slip-knot is the only method of ligature shown and the treatment of hæmophilic bleeding is ignored. The superficial nature of the work is indicated by the fact that the whole subject of malignant disease receives three pages only, and diathermy is the sole method of treatment described. The illustrations are such as are usual in text-books : figures 16 and 27 are identical, and eight of the remaining thirty-five are from instrument catalogues. They are well reproduced, though the coloured frontispiece "Vincent's disease" is (*pace* the artist) astonishingly unlike the usual clinical appearance. Paper and type are excellent, and the whole get-up of the book worthy of the publisher, but it is hard to conceive the reader who will find it of any value.

**Physics for Medical Students.** By J. S. ROGERS, B.A., M.Sc. Pp. x., 205. Illustrated. Melbourne : Melbourne University Press. 1933. Price 11s. 6d.—This book can hardly be looked on as an initial text-book for medical students ; an elementary knowledge of Physics would be necessary before much that is in it could be satisfactorily appreciated. As the sub-title implies, it consists of a series of chapters covering some of the chief applications of Physics in Medicine, and as such it should be of considerable help to students during the professional years of their curriculum. Almost without exception each chapter is independent of what has gone before, and can be read by itself. The subjects covered include osmosis, the colloidal state, hydrogen ion concentration, blood-pressure, heat gain and loss, cochlear function, optics of the eye and the microscope, high-frequency currents, radiations and radio-activity. The section on the various types of radiations is good, and should prove helpful to one who is studying their applications in diagnosis and treatment. The chapter on blood-pressure might be expanded with advantage. One wonders why the chapter on hydrogen ion concentration should be divorced from the other physico-chemical chapters ; it is to be found between the sections on the microscope and high-frequency currents. A welcome feature, uncommon in books of this size, is a section on the history of Physics ; this, though of necessity brief, cannot fail to add to the interest of the book. In addition, a few references are given at the end of some of the chapters. The book is of handy size, and is well-printed and clearly illustrated.

**The Constitution in Health.** By T. E. HAMMOND, F.R.C.S. Pp. ix., 160. London: H. K. Lewis & Co. Ltd. 1934. Price 7s. 6d.—In this book the author deals with a conception which found more favour in a former time than it does with the present generation. It performs a real service in focusing attention on the fact that whilst bacteria are universally recognized as an important factor in the causation of disease, they are not the sole factor. Some more elusive phenomenon must be found to account for the various abnormalities we witness in our patients. As the author stresses in the last chapter, personal idiosyncrasy and abnormal reaction, whether it be to drugs or infections, would well repay further inquiry. Many of the author's statements challenge criticism, *e.g.*, page 72, "It will eventually be found that too much fresh air is not so beneficial as it is thought." The book suffers also from frequent repetition.

**A Synopsis of Hygiene.** By E. W. C. THOMAS, M.D. Pp. viii., 283. Bristol: John Wright & Sons Ltd. 1934. Price 10s. 6d. As the author says, this book is intended for the final year medical student, and follows closely on the lines of the author's larger *Synopsis of Public Health.* The book is written on the lines of others of this series, and will be found most useful for the student studying for his final qualifying examination. Special attention is given to the services and assistance given by a health department to the general medical practitioner.

**Common Skin Diseases.** By A. C. ROXBURGH, M.D. Second Edition. Pp. xxxii., 369. Illustrated. London: H. K. Lewis & Co. Ltd. 1934. Price 16s.—The appearance of a second edition of this text-book in the short space of two years is ample proof of its popularity, and justifies the good Press notices which the first edition received. The second edition has been increased in size by the inclusion of chapters on congenital affections of the skin, atrophy and sclerosis, vesicular and bullous eruptions, and the erythrodermias. There are also some new diagrams and photomicrographs. At the same time the price has been reduced to 16s. The student will find that reading this text-book, combined with attendance at the out-patient department, will give him a sound general knowledge of dermatology, while the practitioner will find it a valuable help in the diagnosis and treatment of those cases with which he has to deal. The simpler methods of treatment are clearly explained, and the indications for more specialized treatment, *e.g.* X-rays, are given. The book can be recommended as a sound and up-to-date presentation of the subject.

**Notes on Milk.** By T. J. STEWART. Pp. 46. Illustrated. London: H. K. Lewis & Co. Ltd. 1934. Price 1s. 6d.—This little pamphlet contains a number of well-indexed facts relative to milk production and consumption. Ice cream is also included. The practitioner may find it useful for reference as it brings together a number of isolated figures and statements that otherwise entail considerable search.

**Stand Up and Slim Down.** By ETTIE A. HORNIBROOK. Sixth Edition. Pp. xiv., 167. Illustrated. London: William Heinemann Ltd. 1934. Price 6s.—This little book comes under the classification of "popular medicine," hence, presumably, its somewhat frivolous title and crude illustrations. It is addressed to women, and particularly to married women. The exercises given are sound, and should, if indulged in regularly, not only keep the exerciser shapely but healthy as well. The diets are simple and, if anything, err on the inadequate side. A big point is rightly made of taking plenty of fluid during the day. For those women inclined to indigestion, adiposity, or general lack of muscle tone, this book can be recommended. There is a full index and the printing is good. It is a pity to have bound it in paper.

**Manipulative Treatment for the Medical Practitioner.** By T. MARLIN, M.D. Pp. vii., 133. Illustrated. London: Edward Arnold & Co. 1934. Price 10s. 6d.—There is a tendency among members of the medical profession to relegate manipulative treatment to the realms of quackery, and having done so, to try to forget its existence. There can be no doubt that a certain type of patient does benefit from this form of treatment, and it is given by osteopaths for conditions of widely different character, with varying results. It is important to realize that many of the diseases for which manipulative treatment is given have nothing to do with the bones or joints, and it is probably this fact that makes it appear to be a quack remedy. Dr. Marlin has taken considerable pains, not the least of which was a journey to America, to go into this matter exhaustively, and in this little book we glean the results of his experience. He shows that manipulation does come into the field of legitimate therapeutics, and obviously it is much more appropriately carried out by qualified medical men than by those who may adopt the method for the cure of carcinoma of the colon. If doctors are able to bring about some of the miraculous cures now credited to the quack, it will undoubtedly make some impression upon the minds of the lay public, and the author shows us the way. In this clearly written and well illustrated work Dr. Marlin has made a valuable contribution to medical literature, which, with careful study and application, should endow its owner with the necessary knowledge and skill to bring about cures in certain types of difficult patients to their mutual benefit.

**Abscess of the Brain: its Pathology, Diagnosis and Treatment.** By E. M. ATKINSON, F.R.C.S. Pp. x., 289. Illustrated. London: Medical Publications Ltd. 1934. Price 21s.—This monograph is based on the author's experience of twenty-three cases of otogenous brain abscess of which eleven were operated on, with seven recoveries. The difficulties with which the surgeon has to contend are well illustrated by these figures, and the author remarks that this "is a very satisfactory recovery rate according to modern statistics." A full and clear account of the pathology of abscess is given, to which subject the author has himself made valuable contributions. Local and general physical signs and diagnosis are described at length, and Mr. Atkinson avoids the error of suggesting that any part of this subject is easy. In treatment he advocates a rather small skin incision and bone removal; multiple punctures of the dura to locate the abscess; a large dural opening and two-tube drainage; and subsequent irrigation of the cavity. In fact, although constantly decrying excessive activity in after-treatment, he is certainly not a purist of the "leave well alone" school. The book is handsomely printed in thick paper with numerous coloured and other plates, and forms a most valuable addition to the study of this intricate and important subject.

**Advice to the Expectant Mother.** Third Edition. By F. J. BROWNE, M.D., D.Sc. Pp. 48. Edinburgh: E. & S. Livingstone. 1934. Price 6d.—In the third edition of Professor Browne's little handbook, *Advice to the Expectant Mother on the Care of Her Health*, the subject-matter has been brought up to date, thus enhancing its value for the class for whom it has been written. The chapter on the common disorders of pregnancy is particularly good. Without being unduly alarming, the author gives a concise account of the serious complications which may arise during gestation, and impresses upon his readers the danger of delay in getting medical help when symptoms pointing to toxæmic conditions arise. The chapter dealing with the hygiene of pregnancy is also excellent, and will be read with benefit by all expectant mothers, but in the chapter devoted to the management of the infant there are one or two statements which are open to criticism. The mother who is breast feeding is advised to nurse from alternate breasts instead of both breasts at each feed, the latter method is usually found to produce a more regular and satisfactory milk supply. The author recommends castor oil as an antidote to the digestive upsets of the overfed infant. It is open to question whether castor oil should not be entirely eliminated from the list of drugs to be used during infancy. It is not generally agreed that a severe loss of weight during the first week of the infant's life should be considered inevitable. With these exceptions this little book can be highly recommended.

**Vaccine Therapy in Respiratory Infections.** By H. T. Gillett, M.D. Pp. xv., 103. Illustrated. London: H. K. Lewis & Co. Ltd. 1933. Price 5s.—Dr. Gillett is to be congratulated upon giving the general practitioner a reliable and well-balanced short work on the subject. The author insists on the importance of the individual needs in each case, deprecating routine methods. The description of the cultuie of the organism and preparation of the vaccines is sound, and his advice as to dosage and remarks on the patient's response are practical and helpful. The author favours the control of frequency and size of the dose in accordance with the response of the patient to each dose, rather than following a rigid course of increasing dosage. The chapter on catarrh and the sequelæ of influenza is particularly helpful, and his reference to the now accepted view of the defensive action of tonsil and adenoid tissue is well worth noting; and the advisability of immunizing the patient after and often before removal is suitably dealt with. In those cases where catarrhal conditions persist after excision of tonsils and adenoids, and where "glands" do not clear up, the condition is due, he points out, to a latent septic focus, which re-infects some remaining lymphoid tissue. Such cases, as a rule, clear up entirely with a course of suitable vaccines, provided there is efficient drainage of any infected accessory sinuses. Dr. Gillett advocates the use of quite small doses of vaccine in all acute conditions, and this is the trend of opinion of most vaccine workers of to-day. From experience we fully endorse the author's statement that provided an efficient vaccine is used in suitable doses at correct intervals good results are assured in chronic and acute infections. This small volume of one hundred pages, including many charts and lists of references, is very well bound and printed, and might with advantage be added to the library of the young general practitioner.

**Pocket Medical Dictionary.** By GEORGE M. GOULD, A.M., M.D. Tenth Edition. London: H. K. Lewis & Co. Ltd. 1934. Price 10s. 6d.—The popularity of this medical dictionary has called for a revision of the tenth edition. Actually there have been sixty-eight printings of Gould's pocket dictionary, aggregating over 800,000 copies. The relegation of the tables of arteries, bones, etc., to an Appendix at the end is a very welcome improvement. Apart from this, no substantial changes have been made. Gould's original plan is adhered to of selecting for inclusion such new words as are of sound and permanent value. This is one of the chief causes of the continued success of his medical dictionary.

